# Prevalence Study of Yaws in the Democratic Republic of Congo Using the Lot Quality Assurance Sampling Method

**DOI:** 10.1371/journal.pone.0006338

**Published:** 2009-07-22

**Authors:** Sibylle Gerstl, Gédeon Kiwila, Mehul Dhorda, Sylvaine Lonlas, Mark Myatt, Benoît Kebela Ilunga, Denis Lemasson, Elisabeth Szumilin, Philippe J. Guerin, Laurent Ferradini

**Affiliations:** 1 Epicentre, Paris, France; 2 Médecins sans Frontières-France, Paris, France; 3 Division of Epidemiology, Institute of Ophthalmology, University College London, London, United Kingdom; 4 Ministry of Health, Kinshasa, Democratic Republic of the Congo; Swiss Paraplegic Research, Switzerland

## Abstract

**Background:**

Until the 1970s the prevalence of non-venereal trepanomatosis, including yaws, was greatly reduced after worldwide mass treatment. In 2005, cases were again reported in the Democratic Republic of the Congo. We carried out a survey to estimate the village-level prevalence of yaws in the region of Equator in the north of the country in order to define appropriate strategies to effectively treat the affected population.

**Methodology/Principal Findings:**

We designed a community-based survey using the Lot Quality Assurance Sampling method to classify the prevalence of active yaws in 14 groups of villages (lots). The classification into high, moderate, or low yaws prevalence corresponded to World Health Organization prevalence thresholds for identifying appropriate operational treatment strategies. Active yaws cases were defined by suggestive clinical signs and positive rapid plasma reagin and Treponema pallidum hemagglutination serological tests. The overall prevalence in the study area was 4.7% (95% confidence interval: 3.4–6.0). Two of 14 lots had high prevalence (>10%), three moderate prevalence (5–10%) and nine low prevalence (<5%.).

**Conclusions/Significance:**

Although yaws is no longer a World Health Organization priority disease, the presence of yaws in a region where it was supposed to be eradicated demonstrates the importance of continued surveillance and control efforts. Yaws should remain a public health priority in countries where previously it was known to be endemic. The integration of sensitive surveillance systems together with free access to effective treatment is recommended. As a consequence of our study results, more than 16,000 people received free treatment against yaws.

## Introduction

Yaws is a communicable, non-venereal treponematosis caused by the bacteria *Treponema pallidum* subsp. *pertenue*. There are two other types of non-venereal endemic treponematosis: endemic syphilis (caused by *Treponema pallidum* subsp. *endemicum*), which is mainly observed in some drier parts of Africa and Asia and may be transmitted by shared use of eating and drinking utensils as well as by skin-to-skin contact, and pinta (caused by *Treponema carateum*), which exists only in parts of Latin America. The three non-venereal endemic treponematoses diseases and venereal syphilis (caused by *Treponema pallidum* subsp. *pallidum*) can only be distinguished by epidemiological characteristics and clinical manifestations, as the commonly used serological tests can not discriminate one disease from the others. Yaws is the most widespread non-venereal treponematosis and is known to be endemic in the Equator province of Democratic Republic of the Congo (DRC) [1]. The disease is transmitted from person to person by direct skin-to-skin contact. It predominantly affects children under 15 years of age in the most underprivileged, remote rural communities. Favourable climate conditions such as humidity and a constant warm temperature appear to be especially important factors for yaws to flourish [2]–[4].

The evolution of yaws is revealed in three stages of clinical manifestations [2]. Early yaws encompasses both the primary and secondary stages (active yaws cases). During the primary stage (3–4 weeks incubation), patients display papillomatous raspberry-like lesions located mainly on the feet and legs. They also have ulcerations of variable size on other sites of the body that are highly contagious. Patients may spontaneously recover from the primary stage or progress to the second stage. The secondary stage consists of several episodes, lasting three to six months, of both contagious and non-contagious lesions. These primary and secondary stages usually last up to five years from the time of infection, with periods of latency in between symptomatic episodes [3]. About 10% of patients with untreated secondary stages pass into the tertiary stage (late yaws), which can occur up to 15 years after the secondary stage [5]. Late lesions may involve the skin and subcutaneous tissues, the mucosa, the bones, and the joints. Tissue destruction is common and characteristic. Third stage lesions are not contagious but disable people in their daily activities [2], [3], [6]. There is evidence that potential sequelae of latent yaws include congenital, visceral, and tertiary central nervous system lesions and may lead to significant osseous, neurological, and ophthalmologic complications [7], [8]. Since the 1940s, a single intramuscular injection of long acting benzathine penicillin has been successfully used for treatment [1], [9].

In the early 1950s an estimated 50 to 100 million people were infected in tropical areas particularly in Africa, Southeast Asia and South America [5]. Following a decision of the Second World Health Assembly in 1949, huge mass treatment campaigns against endemic treponematosis were undertaken in all affected countries with the support of the World Health Organization (WHO). The goal was to eradicate this disease. More than 50 million cases and their direct contacts were treated and, as a result, the prevalence of yaws decreased dramatically to an estimated one to two million cases in the 1980s [10]. Since then, yaws has not been considered a health priority and it is no longer a reportable disease.

However, difficulties encountered by many countries in integrating continued control measures into local health services have led to a gradual build-up and extension of the treponemal reservoir. Thus, after enthusiastic mass treatment campaigns to eradicate yaws until mid-1960s, there has been a strong recrudescence of this disease in the last decades [11]–[13]. Today the situation is particularly precarious in the Sahelian region and western and central Africa where untreated reservoirs of infection are increasing [14]–[16]. The distribution of yaws is closely related to poverty and isolation from organized social and health services, particularly in rural tropical areas [3].

In DRC yaws was thought to be eradicated in the early 1960s. However, in 1975, a study revealed yaws cases among the pygmies in the north of DRC, one of the socio-economically most disadvantaged populations, with up to 90% of the population showing serological evidence of infection at that time [17]. Furthermore, in 1981 endemic foci of yaws with a prevalence of 8% were also identified in the north of DRC [18]. Between 1983 and 1988 almost 2500 yaws cases were reported to WHO [19].

**Figure 1:**
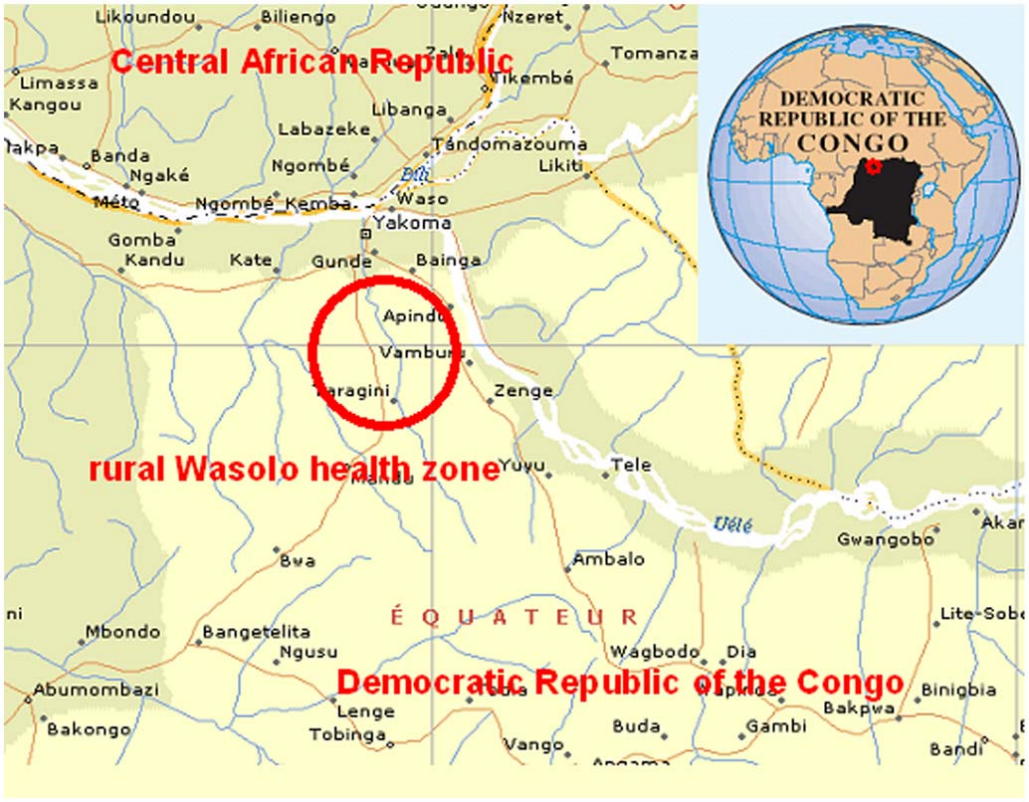
Map of the rural Wasolo health zone, Equator province, Democratic Republic of the Congo, February 2005.

In early 2005, an increased number of yaws cases confirmed through clinical screening were reported from the rural Wasolo health zone, a very remote and isolated region in the north of DRC Equator province (Figure 1). The Ministry of Health and local authorities asked Médecins sans Frontières (MSF) to investigate the yaws prevalence and to identify appropriate treatment strategies. Currently, three different treatment strategies are recommended by WHO according to the observed prevalence of the disease in affected populations: (1) total mass treatment for high prevalence (hyperendemic, over 10%); (2) treatment of active cases, their direct contacts and all children under 15 years (juvenile mass treatment) for medium prevalence (mesoendemic, between 5 and 10%); and (3) treatment of both active cases and direct contacts (selective mass treatment) for low prevalence (hypoendemic, under 5%) areas [2].

MSF and Epicentre conducted a study to estimate the village-level prevalence in the population of the rural Wasolo health zone using Lot Quality Assurance Sampling (LQAS) [20] with the aim of treating the affected population appropriately. The overall prevalence of active yaws in the rural health zone was also estimated.

## Methods

### Study design, setting and population

Between February 16 and March 1, 2005, we carried out a community-based survey in the rural Wasolo health zone, Equator province (Figure 1), after approval from the Ministry of Health in DRC. The population of approximately 32,000 inhabitants [21] lived in 35 villages all situated close to the only road in the area.

All persons older than 6 months living in the 35 villages on the first day of the survey were eligible for inclusion. Children under 6 months of age were excluded for ethical reasons (e.g. taking blood samples under basic field conditions). Written, informed consent was obtained from all participants or his/her legal guardian before inclusion.

### Prevalence of active yaws cases

We used the LQAS method to classify the prevalence of active yaws in groups of villages (lots). The LQAS method has been shown to be useful in identifying areas with high prevalence of disease [22]–[26]. We used two LQAS sampling plans to classify prevalence into three categories [24]–[26]: *n_1_* = 84, *d_1_* = 7 and *n_2_* = 84, *d_2_* = 3. The prevalence in each lot was classified as follows:

*d*>7 classify prevalence as “high”

*d* between 4 and 7 classify prevalence as “moderate”

*d* between 0 and 3 classify prevalence as “low”

where *d* corresponds to the number of active yaws cases found in the lot sample. The classifications of “high”, “moderate”, and “low” correspond to the WHO prevalence thresholds for identifying appropriate public health interventions and operational treatment strategies [2].

**Figure 2:**
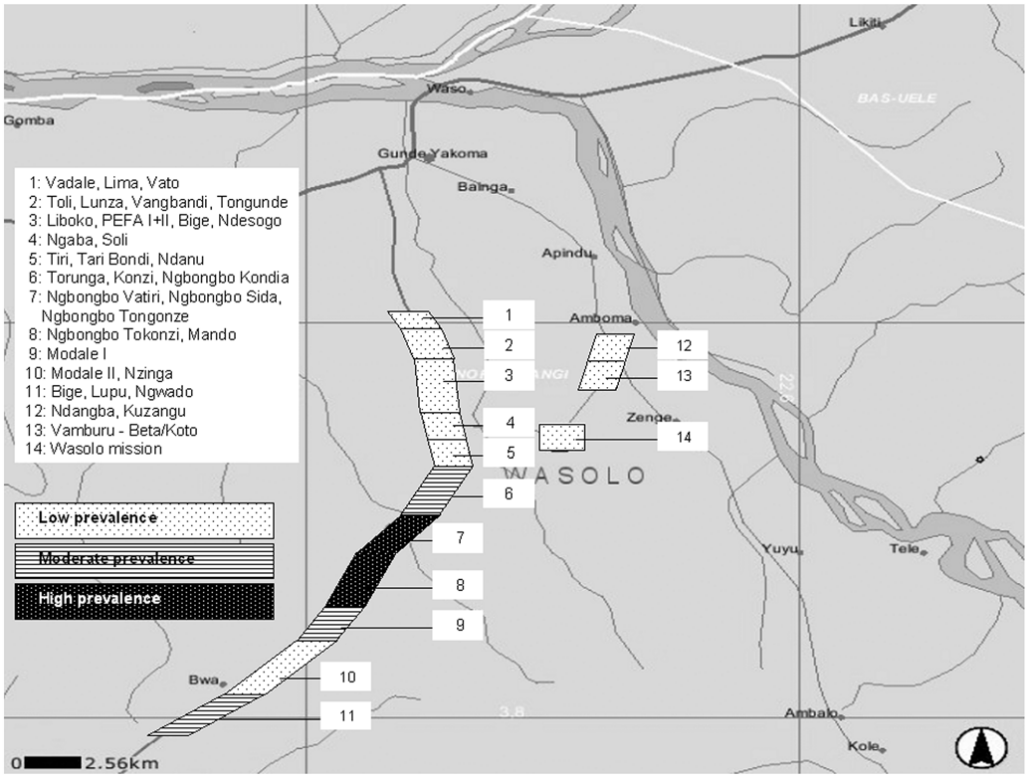
Geographical distributions of active yaw cases per lot and classification of yaws prevalence, rural Wasolo health zone, Equator province, Democratic Republic of the Congo, February 2005.

To ensure lots had similar population sizes, we grouped between one to five villages together according to their geographical proximity, natural boundaries such as rivers or fields and population size (Figure 2). Villages with a small population were more likely to be grouped together than larger villages. We defined a total of 14 lots.

We developed a systematic sampling scheme to randomly select 84 households (defined as a group of people living together and eating from the same pot) from each lot. The size of the sample taken from each village was weighted by the village population so that larger villages contributed more to the lot sample than smaller villages. A uniform sampling fraction was used. The overall lot sample was, therefore, a proportionate, stratified sample [26], [27]. A complete sample (n = 84) was taken from each lot.

As yaws is known to cluster in households, we decided to select only one person per household for participation in the study. This simplified sampling because it allowed households, rather than individuals, to be sampled. This approach has been validated previously in LQAS surveys of trachoma prevalence [24].

All of the villages in the survey area consisted of a single ribbon of houses situated along a road. This made it possible to use a straightforward systematic sampling method to select households. The number of households in the village was estimated by counting doors. A sampling interval was calculated as the ratio of the estimated number of households to the required sample size in the village.

One end of the village was selected by tossing a coin. A random number between one and the sampling interval (inclusive) was read from a table of random numbers. Study teams walked towards the opposite end of the village, counting the houses that they passed, sampling the house indicated by the random number. Subsequent houses were sampled by repeated application of the sampling interval. Sampling stopped when the surveyors reached the calculated number of households for that village.

When a house was sampled, household members were listed and assigned a sequential number. One household member was then sampled at random using a random number table.

The day before study teams arrived in the villages, we visited the chief of the village to seek his permission to survey and to sensitise the population to be present on the day of the study. In the very few cases that households were empty and household members could not be located within a reasonable amount of time, the nearest household was sampled.

### Study procedure

For the detection of yaws lesions, the study population was examined following a standardized checklist for clinical signs suggestive of yaws. Primary yaws chancre was defined as chronic non-genital, painless, non-tender papule (mother yaw), which bursts open and may present as a traumatic ulcer with raised margin located on the legs, feet or buttocks. Secondary lesions, which might be concomitant to the primary lesion, included painful raspberry-like cutaneous lesions (papilloma and papules) in various numbers eventually affecting palms or soles, and painful tender osteoperiostitis affecting the fingers (polydactilitis), nose (“goundou”), tibia or forearm. Tertiary late lesions were defined as mutilating facial ulcer around the nose (“gangosa”), skin gummata, hyperkeratosis of the palms or soles, juxta-articular nodules, or bone deformity including sabre shin tibia. Four teams of three persons each (medical officer, nurse and assistant) carried out the field investigation under the supervision of both an epidemiologist and a dermatologist. Each team took between three and four days to finish one lot. Prior to initiating the survey, the teams received two days of on-site training with special focus on the clinical diagnosis using illustrative pictures and including half a day to pilot the questionnaire.

In the case of clinical suspicion of yaws, individuals or their caretakers/parents if the individual was younger than 15 years were asked about the duration of the lesions and a blood sample (5 ml) was collected for rapid plasma reagin (RPR) and *Treponema pallidum* hemagglutination (TPHA) tests. Immediate treatment with intramuscular long-acting penicillin was given according to WHO recommendations. To reduce pain of intramuscular injections, diluted Lidocaine hydrochloride 1% (1/4) was systematically added to the treatment [28].

Blood samples were centrifuged and stored at 2–8°C. Using a cold chain they were sent to the MSF laboratory in Botetenza (DRC) where an MSF laboratory technician performed all RPR (non-treponemal antigen, RPR-nosticon® II, Biomérieux, Holland) tests. For all RPR positive blood samples, a TPHA (treponemal antigen, Syphilis TPHA liquid®, Human, Germany) test was performed afterwards.

### Case ascertainment

Participants were defined as having active yaws if they presented with cutaneous lesions clinically suggestive of yaws and scored positive on both the non-specific RPR test and the specific TPHA test.

### Statistical analysis

Data were entered into the database using Epi-Data 3.0 software (The EpiData Association, Odense, Denmark). Double data entry and cleaning were performed to check for inconsistencies and errors were corrected. The analysis was done using Stata 8.0 (Stata Corporation, College Station; Texas, USA). In order to calculate the overall prevalence of yaws in the study population, the number of cases in the different lots was weighted according to each lot's population size and combined. Symmetric confidence intervals (95%CI) were calculated after weighting according to each lot's population size. All data presented take into account weighting if required.

## Results

None of the selected households refused to participate in the study. Demographic characteristics of the study population (n = 1176) are shown in Table 1.

**Table 1:** Demographic characteristics of the study population, persons with cutaneous lesions clinically suggestive of yaws and with active yaws confirmed by RPR and TPHA testing, rural Wasolo health zone, Equator province, Democratic Republic of the Congo, February 2005.

	Study population	Persons with cutaneous lesions clinically suggestive of yaws	Persons with active yaws confirmed by RPR* and TPHA† testing
	N = 1176	N = 383	N = 55‡
Median age in years (IQR#)	23 (9–45)	22 (8–45)	21 (10–55)
Range (Minimum, Maximum)	1, 80	7, 72	2, 72
<5 (%)	146 (12.4)	50 (13.1)	3 (5.6)
5–14 (%)	249 (21.2)	94 (24.5)	19 (34.5)
15–29 (%)	287 (24.4)	86 (22.5)	7 (12.7)
30–45 (%)	186 (15.8)	54 (14.1)	7 (12.7)
≥45 (%)	292 (24.8)	99 (25.8)	19 (34.5)
Unknown (%)	16 (1.4)		–
Sex ratio (male/female)	1.2 (643/526)§	1.6 (236/146)±	1.8 (35/20)
Median household size [IQR#]	7 [5]–[9]	7 [5]–[9]	7 [5]–[9]
Main household activity			
Farming (%)	852 (72.4)	281 (73.4)	42 (76.4)

* Rapid Plasma Reagin test.

† *Treponema pallidum* hemagglutination test.

‡ 1 missing data for all listed variables.

# Inter-quartile range.

§ 7 missing data for sex.

± 1 missing data for sex.

### Prevalence of active yaws in the identified lots

Two of 14 lots presented more than seven confirmed active yaws cases, indicating a high prevalence of over 10%. In these lots, total mass treatment was the chosen treatment strategy. For three lots, the number of confirmed active yaws cases was between four and seven (inclusive), indicating a moderate prevalence of 5–10%. Here, juvenile mass treatment was chosen as the treatment strategy. For the remaining nine lots, the number of confirmed active yaws cases was three or lower, indicating a low prevalence of below 5%. Selective mass treatment was chosen as the treatment strategy (Table 2). Figure 2 shows the geographical distribution of all investigated lots with their corresponding prevalence of active yaws.

**Table 2:** Distribution of active yaw cases per lot and classification of yaws prevalence, rural Wasolo health zone, Equator province, Democratic Republic of the Congo, February 2005.

Study lot Village name number		Number of active yaws cases			Prevalence	Treatment strategy used§
		suspected		confirmed		
		clinically*	+ RPR†	+ RPR + TPHA‡		
1	Vadale, Lima, Vato	15	7	3	low	SMT
2	Toli, Lunza, Vangbandi, Tongunde	18	7	1	low	SMT
3	Liboko, PEFA I+II, Bige, Ndesogo	22	13	2	low	SMT
4	Ngaba, Soli	15	10	0	low	SMT
5	Tiri, Tari Bondi, Ndanu	39	21	3	low	SMT
6	Torunga, Konzi, Ngbongbo Kondia	33	25	4	medium	JMT
7	Ngbongbo Vatiri, Ngbongbo Sida, Ngbongbo Tongonze	36	18	11	high	TMT
8	Ngbongbo Tokonzi, Mando	36	26	15	high	TMT
9	Modale I	24	11	4	medium	JMT
10	Modale II, Nzinga	20	6	1	low	SMT
11	Bige, Lupu, Ngwado	42	28	6	medium	JMT
12	Ndangba, Kuzangu	14	9	3	low	SMT
13	Vamburu - Beta/Koto	33	25	1	low	SMT
14	Wasolo mission	36	17	2	low	SMT
	TOTAL	383	223	56		

* Diagnosed clinically.

† Rapid Plasma Reagin test.

‡ *Treponema pallidum* hemagglutination test.

§ TMT (total mass treatment); JMT (juvenile mass treatment, treatment of active cases, their direct contacts and all children under 15 years); SMT (selective mass treatment, treatment of both active cases and direct contacts).

### Overall prevalence of active yaws in the study population

Among 1176 persons examined, 383 (32.6%) presented with cutaneous lesions clinically suggestive of yaws and were tested by RPR. Of these, 223 (58.2%) were RPR positive. Out of the 223 RPR positive individuals, 56 (25.1%) were TPHA positive and defined as having truly active yaws. The overall prevalence of active yaws in the rural Wasolo health zone was 4.7% (95%CI: 3.4–6.0, 56/1176). When separated in two age groups, the overall prevalence of active yaws was 6.1% (95%CI: 3.4–8.9, 22/395) for persons less than 15 years of age and 4.0% (95%CI: 2.6–5.5, 33/765) for those being 15 years and older (age data missing for 16 study participants in the denominator and one case in the numerator).

### Cases with cutaneous lesions clinically suggestive of yaws

Among the 383 persons with cutaneous lesions clinically suggestive of yaws, 37.6% (144/383) were less than 15 years of age (Table 1). Twenty-six individuals (6.8%) displayed lesions suggestive of primary cutaneous yaws, 310 (80.9%) had lesions suggestive of secondary yaws and 17 (4.4%) had lesions suggestive of tertiary cutaneous yaws. The remaining 30 persons showed lesions suggestive of a combination of primary, secondary and tertiary yaws stages.

### Active yaws cases confirmed by RPR and TPHA testing

**Figure 3:**
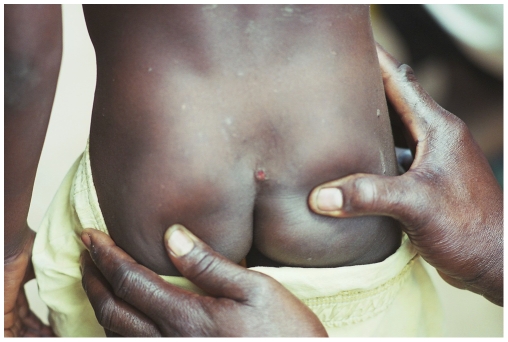
Primary ulcer lesion observed during yaws outbreak, rural Wasolo health zone, Equator province, Democratic Republic of the Congo, February 2005 (picture taken by Laurent Ferradini).

Fifty-six persons had concomitant RPR and TPHA positive tests confirming active yaws. Detailed information was obtained for 55 of them (Table 1). Among those, 40.1% (22/55) were less than 15 years of age. Three persons (5.5%) displayed primary cutaneous yaws lesions, six (10.9%) had primary and secondary yaws lesions and 41 (74.5%) had secondary yaws lesions. One patient displayed pseudo-rachitic curvature of the tibias (Tibial sabre deformity) and juxta-articular nodules and four patients displayed hyperkeratosis on the soles, indicating the tertiary stage. Figures 3–6 show examples of yaws lesions observed. The median self-reported duration of primary cutaneous lesions was 1.0 year (inter-quartile range (IQR): 0.2–1.8), of secondary lesions, 1.5 years (IQR: 0.4–10.0) and of tertiary lesions, 6.5 years (IQR: 2.3–17.0).

**Figure 4 pone-0006338-g004:**
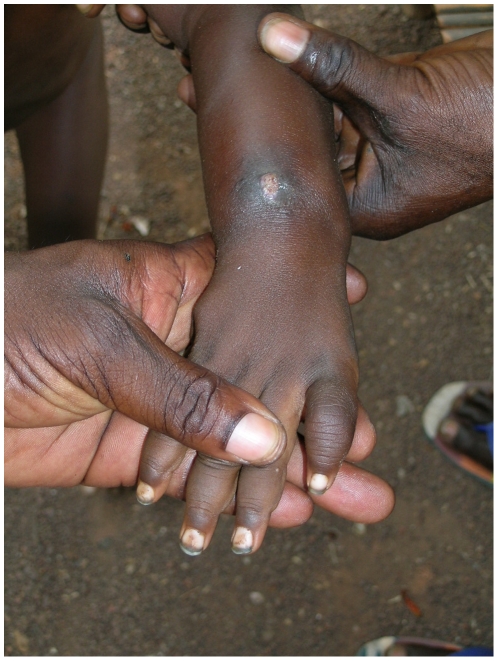
Papilloma secondary lesion with polydactilitis observed during yaws outbreak, rural Wasolo health zone, Equator province, Democratic Republic of the Congo, February 2005 (picture taken by Laurent Ferradini).

**Figure 5 pone-0006338-g005:**
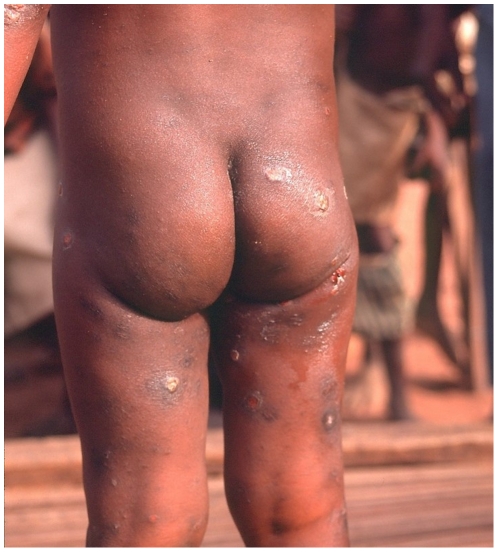
Disseminated papilloma and ulcerative secondary lesions observed during yaws outbreak, rural Wasolo health zone, Equator province, Democratic Republic of the Congo, February 2005 (picture taken by Sibylle Gerstl).

**Figure 6:**
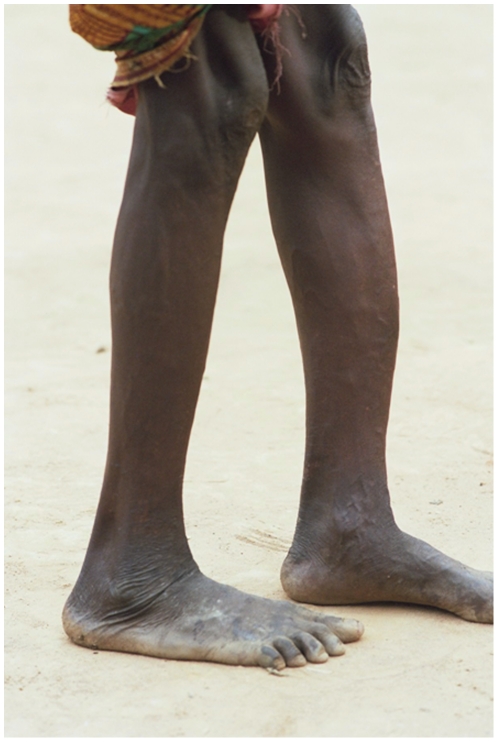
Sabre tibia deformity (tertiary lesions) observed during yaws outbreak, rural Wasolo health zone, Equator province, Democratic Republic of the Congo, February 2005 (picture taken by Laurent Ferradini).

## Discussion

Knowledge of the prevalence of yaws is critical to plan appropriate treatment strategies according to current WHO recommendations. As it is well-known that the disease is clustered and that its prevalence can vary greatly between villages, we used the LQAS method as a rapid way of assessing the prevalence in groups of villages instead of using classical sampling in the entire population. The finding that some lots had a high prevalence of over 10% while the overall prevalence was 4.7% underlines the benefit of this strategy. If the treatment decision had been based solely on overall prevalence (defined as ‘moderate’ in our study), total mass treatment would not have been carried out in any of the villages, even though some displayed high prevalence of the disease. In addition, when considering only the overall prevalence estimation, it would have been difficult to follow treatment recommendations because the 95% confidence interval of 3.4 to 6.0 encompassed the critical 5% threshold level recommended by WHO to define appropriate distinct treatment strategies.

In April 2005 following this study, MSF launched a campaign offering free treatment to more than 16,000 people in the villages of the rural Wasolo health zone based on the prevalence estimates in the different groups of villages. Our study nicely illustrates the benefit of using LQAS as an operational research method to rapidly determine the prevalence of yaws in distinct areas in order to define appropriate treatment strategies.

Some data and methodological limitations of our study should be mentioned. The different study teams had varying levels of clinical skills in diagnosing the disease. In the context of a known yaws outbreak, clinical diagnosis may lead to an overestimation of potential active yaws cases. Systematic serological tests of suspected cases were performed to confirm the diagnosis and illustrated this overestimation problem. A relatively low percentage of suspected cases were confirmed serologically. For instance, lesions of the soles, which are very frequent in rural populations not wearing shoes, where most commonly clinically overdiagnosed as yaws but not confirmed by RPR and TPHA tests. However, these tests have themselves well-known limitations. Indeed, *Treponema pallidum* includes three subspecies of antigenically highly related organisms, which can only be distinguished on the basis of epidemiological characteristics and clinical manifestations, but not by morphological, immunological and serological methods [29], [30]. Several laboratory methods of identification have been proposed so far, but all have failed to differentiate distinct subspecies [3], [31]–[33]. Thus, the serological tests used in this study could not distinguish between subspecies of *Treponema pallidum* and did not allow us to confidently diagnose yaws over other treponemal infections. However, RPR and TPHA positive tests in persons under 15 years old are very likely to be due to yaws, because this population is usually not yet sexually active and is very unlikely to display syphilis infection. In our study, 40% of persons with active yaws were under 15 years old and thus most likely represent true active yaws cases. Overall prevalence of active yaws cases was also slightly higher for persons under 15 years old than for the entire study population. For persons above 15 years old who are known to be sexually active, syphilis infection cannot be strictly excluded. However, none of these persons had unequivocal lesions suggestive of syphilis, making such a diagnosis unlikely in the presence of lesions classically suggestive of yaws. Therefore, we are confident that the serologically confirmed cases in our study corresponded to true yaws. We are also aware of reported examples of nonsexual transmission of syphilis via direct skin-to-skin contact [34] and this possibility could not be excluded in the context of our study.

Finally, we observed that only about one quarter of the RPR positive cases were confirmed by TPHA. It is known that the RPR test is not specific for antibodies to *Treponema spp*. and that some concomitant diseases such as malaria and HIV infections may cause false-positive tests [34], [35]. Since there was no prevalence data available for other diseases in our study area, we do not have any satisfactory explanations for the high rate of false positives RPR tests observed within the context of our study.

Between December 2004 and February 2005 more than 2500 cases with cutaneous lesions clinically suggestive of yaws were reported by health personnel working in different health centres in the rural Wasolo health zone, an isolated region in the North of DRC (personal communication, main health office, rural health zone Wasolo, 2005). These findings were confirmed by our survey revealing an overall prevalence of active yaws of 4.7% (95%CI: 3.4–6.0). This prevalence estimate was similar to that reported in the few yaws outbreaks documented in other African countries, such as in Ghana in 1981 (4.0%) [15] and in the mid-Hawal river valley in Nigeria in 1998 (4.2%) [36]. More than 90% of individuals with active yaws in our study presented with the secondary stage and consequently had long-standing contagious lesions. The presence of these secondary stage cases could explain the transmission and maintenance of the disease in the study area, an assumption supported by the characteristics of a yaws outbreak observed in Thailand [37].

The presence of yaws in the rural Wasolo health zone is very likely to be the consequence of a progressive increase of yaws cases in an area where the disease was supposed to be eradicated but where new cases had been reported in the past [18], [19]. This occurred most likely because of the steady degradation of health structures in this very remote, rural area. Indeed, DRC is going through a dreadful civil war and the study area in particular was heavily involved in long lasting armed conflicts during the last few years. The distribution of endemic treponematosis is known to be closely related to poverty and isolation from organised social and health services [3]. As an additional consequence of poor health structures, control of yaws and other diseases became largely inefficient. Furthermore, as in many countries, yaws is no longer considered a health priority in the DRC, explaining why its simple treatment is not currently available even at health centres. However, the International Task Force for Disease Eradication (ITFDE) reviewed the case of yaws at its eleventh meeting in October 2007 and the disease seems to be back on WHO's agenda [29], [38]. In addition, articles in scientific journals on yaws currently raise interest [39], [40].

Among measures for potential eradication of the disease, the implementation of a long term yaws control system with free access to treatment is urgently needed to avoid the further spread of this debilitating disease in the rural Wasolo health zone but also in neighbouring health zones and districts. Horizontal programmes in which community-based health workers are involved in all aspects of disease control have yielded very positive results in Ecuador [41]. In Malaysia, excellent results were obtained from the appropriate training of all health workers to recognise and treat yaws cases [42]. In India, the goal of having zero cases was achieved in 2006 mainly due to active case detection and treatment of cases and contacts [43], [44]. According to a study conducted in Ghana, the most critical requirement might be the availability and affordability of benzathine penicillin in remote health centres [10].

In conclusion, yaws is a highly curable neglected tropical disease. Our study showed the advantage of LQAS as a rather uncommon sampling method to rapidly estimate the prevalence of yaws in order to treat affected populations according to WHO recommendations. The control of yaws should remain a public health priority. The disability and disfigurement resulting from this disease cause individual suffering and will also have indirect and largely unrecorded effects on the morbidity and mortality of affected populations.
